# Age-specific differential changes on gut microbiota composition in patients with major depressive disorder

**DOI:** 10.18632/aging.102775

**Published:** 2020-02-10

**Authors:** Jian-Jun Chen, Sirong He, Liang Fang, Bin Wang, Shun-Jie Bai, Jing Xie, Chan-Juan Zhou, Wei Wang, Peng Xie

**Affiliations:** 1 Institute of Life Sciences, Chongqing Medical University, Chongqing 400016, China; 2 Chongqing Key Laboratory of Cerebral Vascular Disease Research, Chongqing Medical University, Chongqing 400016, China; 3 Department of Immunology, College of Basic Medicine, Chongqing Medical University, Chongqing 400016, China; 4 Department of Neurology, Yongchuan Hospital of Chongqing Medical University, Chongqing 402160, China; 5 Department of Laboratory, The First Affiliated Hospital of Chongqing Medical University, Chongqing 400016, China; 6 Department of Endocrinology and Nephrology, Chongqing University Central Hospital, Chongqing Emergency Medical Center, Chongqing 400014, China; 7 NHC Key Laboratory of Diagnosis and Treatment on Brain Functional Diseases, Chongqing Medical University, Chongqing 400016, China; 8 Department of Neurology, The First Affiliated Hospital of Chongqing Medical University, Chongqing 400016, China

**Keywords:** major depressive disorder, gut microbiota, Firmicutes, Bacteroidetes, Actinobacteria

## Abstract

Emerging evidence has shown the age-related changes in gut microbiota, but few studies were conducted to explore the effects of age on the gut microbiota in patients with major depressive disorder (MDD). This study was performed to identify the age-specific differential gut microbiota in MDD patients. In total, 70 MDD patients and 71 healthy controls (HCs) were recruited and divided into two groups: young group (age 18-29 years) and middle-aged group (age 30-59 years). The 16S rRNA gene sequences were extracted from the collected fecal samples. Finally, we found that the relative abundances of *Firmicutes* and *Bacteroidetes* were significantly decreased and increased, respectively, in young MDD patients as compared with young HCs, and the relative abundances of *Bacteroidetes* and *Actinobacteria* were significantly decreased and increased, respectively, in middle-aged MDD patients as compared with middle-aged HCs. Meanwhile, six and 25 differentially abundant bacterial taxa responsible for the differences between MDD patients (young and middle-aged, respectively) and their respective HCs were identified. Our results demonstrated that there were age-specific differential changes on gut microbiota composition in patients with MDD. Our findings would provide a novel perspective to uncover the pathogenesis underlying MDD.

## INTRODUCTION

Major depressive disorder (MDD) is viewed as a major public health problem globally. MDD has a substantial impact on society and individuals, such as increasing economic burden and decreasing labor productivity [1–3]. At a global level, more than 300 million people are estimated to suffer from MDD, which is equivalent to 4.4% of the world’s population [4]. However, the pathogenesis of MDD is still unclear. Some theories have been developed to explain the biological mechanisms of MDD, such as neurotrophic alterations and neurotransmission deficiency [5, 6]. However, none of these theories has been universally accepted. Therefore, there is a pressing need to identify novel pathophysiologic mechanisms underlying this disease.

In recent years, mounting evidence has shown that gut microbiota could play a vital role in every aspect of physiology [7]. It is the largest and most direct external environment of humans. Previous studies found that the disturbance of gut microbiota had a crucial role in the pathogenesis of many diseases [8–10]. Recent studies reported that gut microbiota could affect the host brain function and host behaviors through microbiota-gut-brain axis [11, 12]. Using germ-free mice, we found that gut microbiota could influence the gene levels in the hippocampus of mice and lipid metabolism in the prefrontal cortex of mice [13, 14]. Our clinical studies demonstrated that the disturbance of gut microbiota might be a contributory factor in the development of MDD [15, 16].

Nowadays, emerging evidence has shown the age-related changes in gut microbiota composition. For example, *Firmicutes* is the dominant taxa during the neonatal period, but *Actinobacteria* and *Proteobacteria* are about to increase in three to six months [17]. While in adults, Vemuri et al. reported that *Bacteroidetes* and *Firmicutes* were the dominant taxa [18]. Meanwhile, compared to younger individuals, the abundance of *Bacteroidetes* is significantly higher in frailer older individuals [19]. These results showed that there was a close relationship between age and gut microbiota composition. Ignoring this relationship would affect the robust of results when exploring the mechanism of action of gut microbiota in diseases. Therefore, to study the relationship between gut microbiota and MDD patients in different age groups, we recruited 52 young subjects aged from 18 to 29 years (27 healthy controls (HCs) and 25 MDD patients) and 89 middle-aged subjects aged from 30 to 59 years (44 HCs and 45 MDD patients). The main purpose of this study was to identify the age-specific differential changes on gut microbiota composition in MDD patients. Our results would display the different changes of gut microbiota composition along with age between HCs and MDD patients.

## RESULTS

### Differential gut microbiota composition

**Figure 1:**
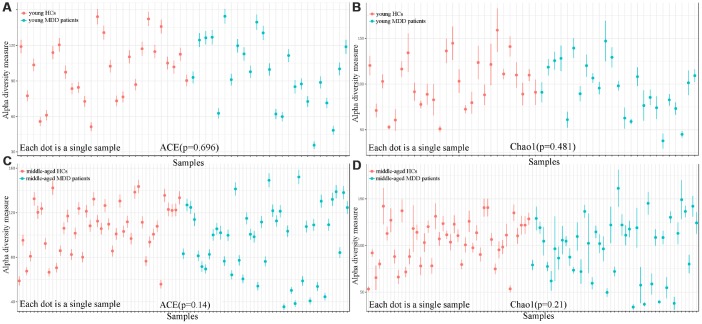
**Comparison of alpha diversity between HCs and MDD patients.** (**A**, **B**) ACE and Chao1 indexes showed no significant differences between young HCs (n=27) and young MDD patients (n=25); (**C**, **D**) ACE and Chao1 indexes showed no significant differences between middle-aged HCs (n=44) and middle-aged MDD patients (n=45).

**Figure 2:**
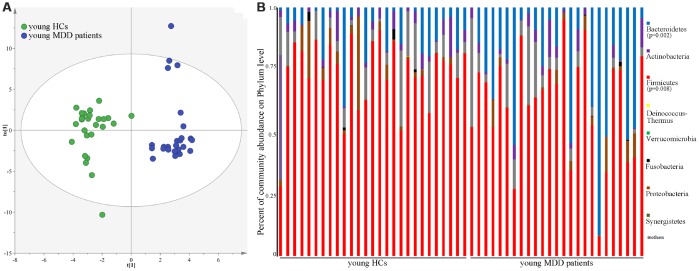
**16S rRNA gene sequencing reveals changes to microbial abundances in young MDD patients.** (**A**) OPLS-DA model showed an obvious difference in microbial abundances between the two groups (HCs, n=27; MDD, (n=25); (**B**) the relative abundances of Firmicutes and Bacteroidetes were significantly changed in young MDD patients (n=25) as compared with young HCs (n=27).

**Figure 3:**
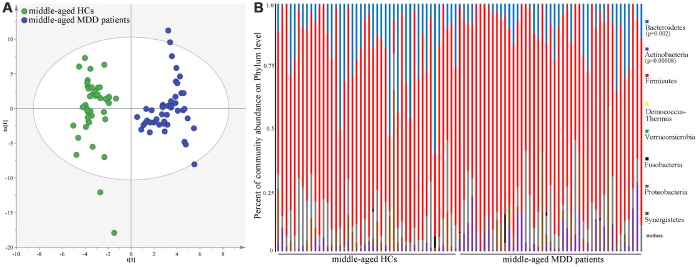
**16S rRNA gene sequencing reveals changes to microbial abundances in middle-aged MDD patients.** (**A**) OPLS-DA model showed an obvious difference in microbial abundances between the two groups (HCs, n=44; MDD, (n=45); (**B**) the relative abundances of *Bacteroidetes* and *Actinobacteria* were significantly changed in middle-aged MDD patients (n=45) as compared with middle-aged HCs (n=44).

As shown in Figure 1, the results of abundance-based coverage estimator (ACE) and Chao1 showed that there was no significant difference in OTU richness between MDD patients (young and middle-aged, respectively) and their respective HCs. However, the OPLS-DA model built with young HCs and young MDD patients showed an obvious difference in microbial abundances between these two groups (Figure 2A). The relative abundances of *Firmicutes* and *Bacteroidetes* were significantly decreased and increased, respectively, in young MDD patients as compared with young HCs (Figure 2B). Meanwhile, the OPLS-DA model built with middle-aged HCs and middle-aged MDD patients showed an obvious difference in microbial abundances between these two groups (Figure 3A). The relative abundances of *Bacteroidetes* and *Actinobacteria* were significantly decreased and increased, respectively, in middle-aged MDD patients as compared with middle-aged HCs (Figure 3B).

### Key discriminatory OTUs

**Figure 4:**
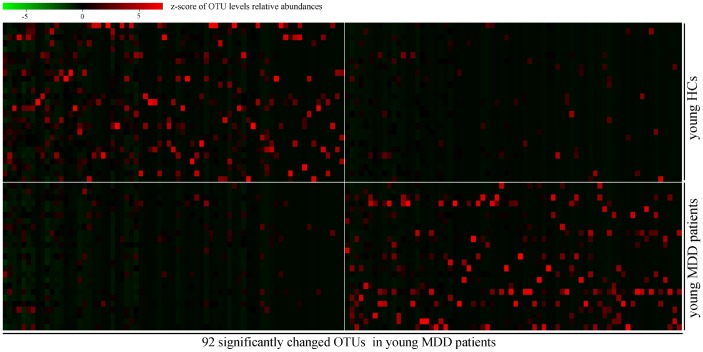
**Heatmap of discriminative OTUs abundances between young HCs (n=27) and young MDD patients (n=25).**

**Figure 5:**
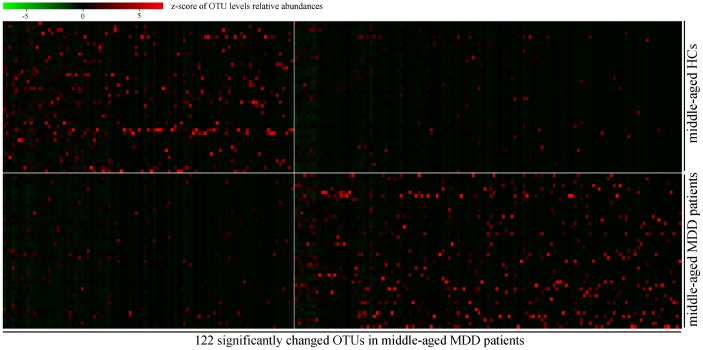
**Heatmap of discriminative OTUs abundances between middle-aged HCs (n=44) and middle-aged MDD patients (n=45).**

In order to find out the gut microbiota primarily responsible for the separation between MDD patients (young and middle-aged, respectively) and their respective HCs, the Random Forests classifier was used. A total of 92 OTUs responsible for the separation between young MDD patients and young HCs were identified (Figure 4). These OTUs were mainly assigned to the Families of Bacteroidaceae, Clostridiaceae_1, Coriobacteriaceae, Erysipelotrichaceae, Lachnospiraceae, Peptostreptococcaceae and Ruminococcaceae. Meanwhile, a total of 122 OTUs responsible for the separation between middle-aged MDD patients and middle-aged HCs were identified (Figure 5). These OTUs were mainly assigned to the Families of Lachnospiraceae, Coriobacteriaceae, Streptococcaceae, Prevotellaceae, Bacteroidaceae, Eubacteriaceae, Actinomycetaceae, Sutterellaceae, Acidaminococcaceae, Erysipelotrichaceae, Ruminococcaceae, and Porphyromonadaceae.

### Differentially abundant bacterial taxa

**Figure 6:**
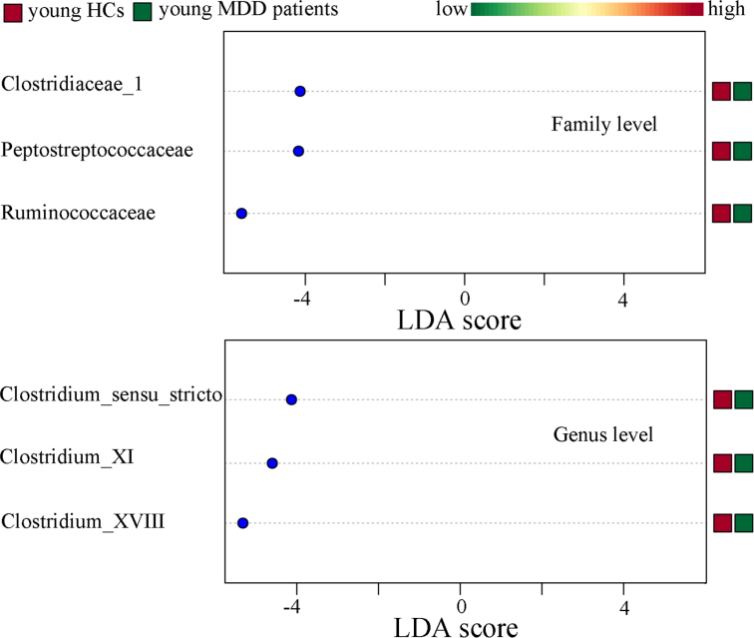
**Differentially abundant features identified by LEfSe that characterize significant differences between young HCs (n=27) and young MDD patients (n=25).**

**Figure 7:**
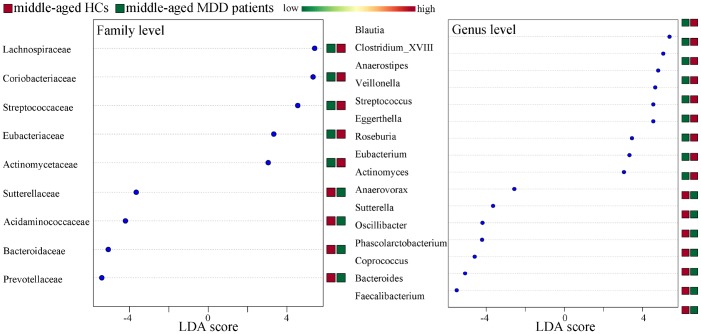
**Differentially abundant features identified by LEfSe that characterize significant differences between middle-aged HCs (n=44) and middle-aged MDD patients (n=45).**

**Figure 8:**
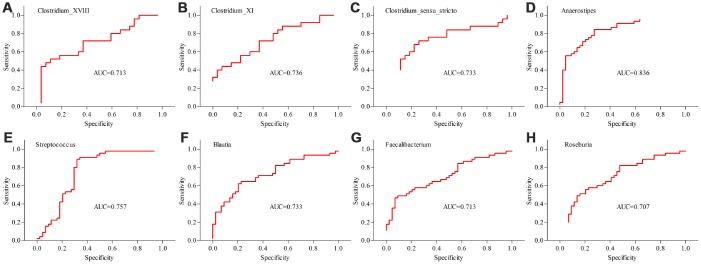
**Differential taxa (at the genus level) with AUC>0.7 in diagnosing MDD patients from HCs.** (**A**–**C**) the diagnostic performances of three taxa in diagnosing young MDD patients (n=25) from young HCs (n=27); (**D**–**H**) the diagnostic performances of five taxa in diagnosing middle-aged MDD patients (n=45) from middle-aged HCs (n=44).

Differentially abundant bacterial taxa responsible for the differences between MDD patients (young and middle-aged, respectively) and their respective HCs were identified by the metagenomic Linear Discriminant Analysis (LDA) Effect Size (LEfSe) approach (LDA score>2.0 and p-value<0.05). In total, six bacterial taxa with statistically significant and biologically consistent differences in young MDD patients were identified (Figure 6). Meanwhile, fifteen bacterial taxa with statistically significant and biologically consistent differences in middle-aged MDD patients were identified (Figure 7). In addition, using the receiver operating characteristic (ROC) curve analysis, we found that Clostridium_sensu_stricto, Clostridium_XI and Clostridium_XVIII showed good diagnostic performance (area under the curve (AUC) >0.7) in diagnosing young MDD patients (Figure 8A–8C). We also found that Anaerostipes, Streptococcus, Blautia, Faecalibacterium and Roseburia showed good diagnostic performance (AUC>0.7) in diagnosing middle-aged MDD patients (Figure 8D–8H).

### Effects of age on microbial abundances

**Figure 9:**
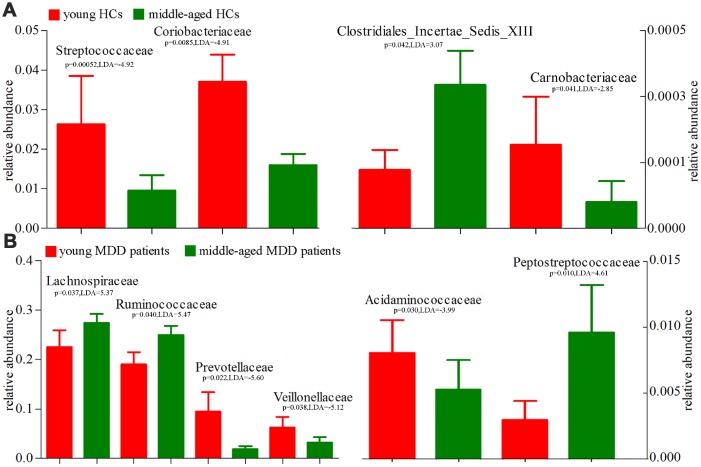
**16S rRNA gene sequencing reveals changes to microbial abundances at family level (Mean±SEM).** (**A**) the abundances of four taxonomic levels were significantly changed between young HCs (n=27) and middle-aged HCs (n=44); (**B**) the abundances of six taxonomic levels were significantly changed between young MDD patients (n=25) and middle-aged MDD patients (n=45).

**Figure 10:**
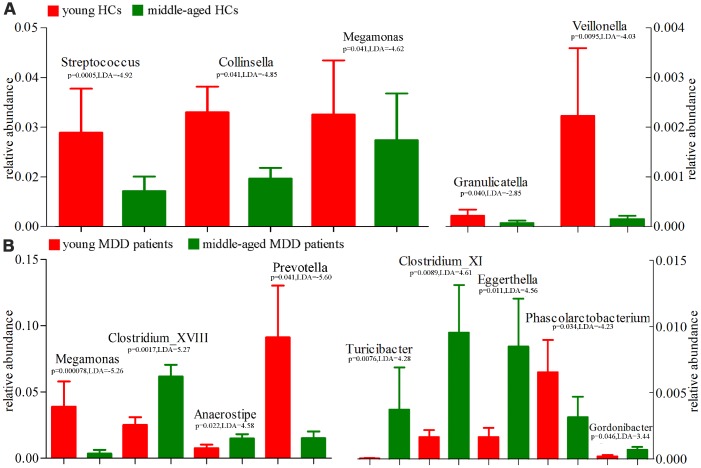
**16S rRNA gene sequencing reveals changes to microbial abundances at genus level (Mean±SEM).** (**A**) the abundances of five taxonomic levels were significantly changed between young HCs (n=27) and middle-aged HCs (n=44); (**B**) the abundances of nine taxonomic levels were significantly changed between young MDD patients (n=25) and middle-aged MDD patients (n=45).

Using the LEfSe approach, we identified four differentially abundant bacterial taxa (the Family level) between young HCs and middle-aged HCs (Streptococcaceae, Coriobacteriaceae, Carnobacteriaceae and Clostridiales_Incertae_Sedis_XIII) (Figure 9A); we also identified six differentially abundant bacterial taxa (the Family level) between young MDD patients and middle-aged MDD patients (Prevotellaceae, Acidaminococcaceae, Veillonellaceae Peptostrep-tococcaceae, Lachnospiraceae and Ruminococcaceae) (Figure 9B). Meanwhile, using the LEfSe approach, we identified five differentially abundant bacterial taxa (the Genus level) between young HCs and middle-aged HCs (Streptococcus, Veillonella, Granulicatella, Collinsella and Megamonas) (Figure 10A). All these bacterial taxa were significantly decreased in middle-aged HCs; we also identified nine differentially abundant bacterial taxa (the Genus level) between young MDD patients and middle-aged MDD patients (Megamonas, Prevotella, Phascolarctobacterium, Anaerostipes, Clostridium_XVIII, Gordonibacter, Eggerthella, Clostridium_XI and Turicibacter) (Figure 10B).

### Effects of medication on microbial abundances

**Figure 11:**
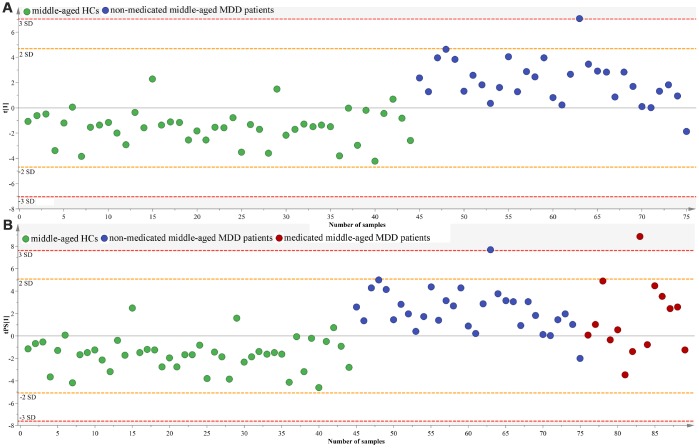
**Assessment of gut microbiota composition in non-medicated and medicated middle-aged MDD patients.** (**A**) middle-aged HCs (n=44) and non-medicated middle-aged MDD patients (n=31) were effectively separated by the built OPLS-DA model; (**B**) 14 medicated middle-aged MDD patients were correctly predicted by the model.

To determinate the homogeneity of gut microbiota composition between medicated and non-medicated MDD patients, we firstly used the middle-aged HCs and non-medicated middle-aged MDD patients to built OPLS-DA model (Figure 11A). The results showed that 41 of the 44 middle-aged HCs and 30 of the 31 non-medicated middle-aged MDD patients were correctly diagnosed by the OPLS-DA model. Then, we used the built model to predict class membership of 14 medicated middle-aged MDD patients. The T-predicted scatter plot showed that 11 of the 14 medicated middle-aged MDD patients were correctly predicted (Figure 11B). These finding indicated that the gut microbiota composition of non-medicated middle-aged MDD patients were distinct from middle-aged HCs, but not from medicated middle-aged MDD patients.

## DISCUSSION

Individuals in the different phases of life cycle (named children, young, middle-aged and elderly) present different biological characteristics and disease risks [20]. Understanding the different characteristics of patients in particular age phases could be facilitated to prevent and treat diseases. According to the World Health Organization reported, the prevalence rates of depression vary by age, peaking in older adulthood. It also occurs in children, but at a lower level compared with older age groups. Here, we conducted this work to investigate how the gut microbiota composition changed in different age phases of MDD patients, and found some age-specific differential gut microbiota in MDD patients. Our results could provide a new perspective on exploring the pathogenesis of MDD.

Many previous studies focused on the effects of gut microbiota on brain functions [21, 22]. However, few studies have taken the effects of age on gut microbiota into consideration when exploring the pathogenesis of MDD. Our previous study found that the relative abundances of *Bacteroidetes* and *Actinobacteria* were significantly decreased and increased, respectively, in MDD patients as compared with HCs [15]. But, in this study, we found that the relative abundances of *Firmicutes* and *Bacteroidetes* were significantly decreased and increased, respectively, in young MDD patients as compared with young HCs, and the relative abundances of *Bacteroidetes* and *Actinobacteria* were significantly decreased and increased, respectively, in middle-aged MDD patients as compared with middle-aged HCs. This disparity might be caused by the different age structures. Meanwhile, only 35 key discriminatory OTUs were significantly changed in both young (92 key discriminatory OTUs) and middle-aged (127 key discriminatory OTUs) MDD patients. Moreover, the differentially abundant bacterial taxa in young and middle-aged MDD patients were totally different at both Family level and Genus level. These results demonstrated that it was necessary to identify the age-specific differential gut microbiota in patients with MDD.

As far as we known, gut microbiota composition and its function could be easily influenced by many factor, such as gender, age, life experiences, dietary habit and genetics. Mariat et al reported that the *Firmicutes/Bacteroidetes* ratio of the human microbiota could change with age [23]. Interestingly, here we found that the relative abundance of *Firmicutes* was significantly decreased in young MDD patients, but not in middle-aged MDD patients; the relative abundance of *Bacteroidetes was* significantly increased and decreased, respectively, in young and middle-aged MDD patients. In our previous studies, we did not analyze the potential effects of medication on gut microbiota composition in MDD patients [15, 16]. Here, due to the small samples of young group, we only used the middle-aged group to analyze the effects of medication on the gut microbiota composition. The results showed that the medication seemed to have little effects on gut microbiota composition in MDD patients. However, our findings had to be cautiously interpreted due to the relatively small samples using to analyze the effects of medication on gut microbiota composition.

The relative abundance of genus Clostridium_XVIII was not found to be significantly different between MDD patients and HCs in our previous study [15]. However, in this study, we found that the relative abundance of genus Clostridium_XVIII was significantly decreased in young MDD patients compared with young HCs, while increased in middle-aged MDD patients compared with middle-aged HCs. The reason of this disparity might be that age could significantly affect the relative abundance of genus Clostridium_XVIII in MDD patients, but not HCs: i) compared to young MDD patients, the middle-aged MDD patients had a significantly higher relative abundance of genus Clostridium_XVIII; and ii) the relative abundance of genus Clostridium_XVIII was similar between young and middle-aged HCs. Meanwhile, we found that the relative abundance of genus Megamonas was significantly decreased in both middle-aged HCs and middle-aged MDD patients compared to their respective young populations. In addition, most of differential bacterial taxa were significantly decreased in middle-aged HCs compared with young HCs, but only about half of differential bacterial taxa were significantly decreased in middle-aged MDD patients compared with young MDD patients. Lozupone et al. reported that gut microbiota could not only simply determine the certain host characteristics, but also respond to signals from host via multiple feedback loops [24]. Therefore, our results suggested that age might have the different effects on the gut microbiota composition of HCs and MDD patients, and should always be considered in investigating the relationship between MDD and gut microbiota.

Limitations should be mentioned here. Firstly, the number of HCs and MDD patients was relatively small, and future works were still needed to further study and support our results. Secondly, we only explored the age-specific differential changes on gut microbiota composition in patients with MDD; future studies should further investigate the functions of these identified differential gut microbiota using metagenomic technology. Thirdly, all included subjects were from the same site and ethnicity; thus, the potential site- and ethnic-specific biases in microbial phenotypes could not be ruled out, which might limit the applicability of our results [25–28]. Fourthly, only young and middle-aged groups were recruited, future studies should recruit old-aged group and children group to further identify the age-specific differential gut microbiota in the different phases of life cycle. Fifthly, we only investigated the differences in gut microbiota between HCs and MDD patients on phylum level, family level and genus level. Future studies were needed to further explore the differences on other levels, such as class level and species level. Sixthly, we did not collect information on smoking, a factor which could influence the gut microbiota composition. Future studies were needed to analyze how the smoking influenced the gut microbiota composition in the different phases of life cycle of subjects. Finally, we found that the medication status of subjects could not significantly affect our results. However, limited by the relatively small samples, this conclusion was needed future studies to further validate.

In conclusion, in this study, we found that there were age-specific differential changes on gut microbiota composition in patients with MDD, and identified some age-specific differentially abundant bacterial taxa in MDD patients. Our findings would provide a novel perspective to uncover the pathogenesis underlying MDD, and potential gut-mediated therapies for MDD patients. Limited by the small number of subjects, the results of the present study were needed future studies to validate and support.

## MATERIALS AND METHODS

### Subject recruitment

This study was approved by the Ethical Committee of Chongqing Medical University and conformed to the provisions of the Declaration of Helsinki. In total, there were 27 young HCs (aged 18-29 years) and 25 young MDD outpatients (aged 18-29 years) in the young group; there were 44 middle-aged HCs (aged 30-59 years) and 45 middle-aged MDD outpatients (aged 30-59 years) in the middle-aged group. Most of MDD patients were first-episode drug-naïve depressed subjects. There were only seven young MDD patients and 14 middle-aged MDD patients receiving medications. The detailed information of these included subjects was described in Table 1. All HCs were recruited from the Medical Examination Center of Chongqing Medical University, and all MDD patients were recruited from the psychiatric center of Chongqing Medical University. MDD patients were screened in the baseline interview by two experienced psychiatrists using the DSM-IV (Diagnostic and Statistical Manual of Mental Disorders, 4th Edition)-based Composite International Diagnostic Interview (CIDI, version2.1). The Hamilton Depression Rating Scale (HDRS) was used to assess the depressive symptoms of each patient, and those patients with HDRS score >=17 were included. Meanwhile, MDD patients were excluded if they had other mental disorders, illicit drug use or substance abuse, and were pregnant or menstrual women. HCs were excluded if they were with mental disorders, illicit drug use or systemic medical illness. All the included subjects provided written informed consent before sample collection.

**Table 1:** Demographic and clinical characteristics of MDD patients and HCs^a^.

	**Young group (18-29 years)**			**Middle-aged group (30-59 years)**		
	**HC**	**MDD**	**p-value**	**HC**	**MDD**	**p-value**
**Sample Size**	27	25	–	44	45	–
**Age (years)^c^**	24.96±2.31	24.0±3.74	0.26	47.16±8.07	44.96±7.76	0.19
**Sex (female/male)**	19/8	18/7	0.89	34/10	31/14	0.37
**BMI**	21.53±2.37	22.13±2.24	0.35	23.23±2.33	22.64±2.64	0.26
**Medication (Y/N)**	0/27	7/18	–	0/44	14/31	–
**HDRS scores**	0.29±0.61	22.64±3.18	<0.00001	0.34±0.74	23.0±4.61	<0.00001

^***a***^Abbreviations: HDRS: Hamilton Depression Rating Scale; HCs: healthy controls; MDD: major depressive disorder; BMI: body mass index.

### 16s rRNA gene sequencing

We used the standard PowerSoil kit protocol to extract the bacterial genomic DNA from the fecal samples. Briefly, we thawed the frozen fecal samples on ice and pulverized the samples with a pestle and mortar in liquid nitrogen. After adding MoBio lysis buffer into the samples and mixing them, the suspensions were centrifuged. The obtained supernatant was moved into the MoBio Garnet bead tubes containing MoBio buffer. Subsequently, we used the Roche 454 sequencing (454 Life Sciences Roche, Branford, PA, USA) to extract the bacterial genomic DNA. The extracted V3-V5 regions of 16S rRNA gene were polymerase chain reaction-amplified with bar-coded universal primers containing linker sequences for pyrosequencing [29].

The Mothur 1.31.2 (http://www.mothur.org/) was used to quality-filtered the obtained raw sequences to identify unique reads [30]. Raw sequences met any one of the following criteria were excluded: i) less than 200bp or greater than 1000bp; ii) contained any ambiguous bases, primer mismatches, or barcode mismatches; and iii) homopolymer runs exceeding six bases. The remaining sequences were assigned to operational taxonomic units (OTUs) with 97% threshold, and then taxonomically classified according to Ribosomal Database Project (RDP) reference database [31]. We used these taxonomies to construct the summaries of the taxonomic distributions of OTUs, and then calculated the relative abundances of gut microbiota at different levels. The abovementioned procedure and most of data were from our previous studies [15, 16].

### Statistical analysis

Richness was one of the two most commonly used alpha diversity measurements. Here, we used two different parameters (Chao1 and ACE) to estimate the OTU richness [32, 33]. The orthogonal partial least squares discriminant analysis (OPLS-DA) was a multivariate method, which was used to remove extraneous variance (unrelated to the group) from the sequencing datasets. The LEfSe was a new analytical method for discovering the metagenomic biomarker by class comparison. The bacterial taxa with LDA score>2.0 were viewed as the differentially abundant bacterial taxa responsible for the differences between different groups. Here, both OPLS-DA [34, 35] and LEfSe were used to reduce the dimensionality of datasets and identify the differentially abundant bacterial taxa (the Family level and Genus level) that could be used to characterize the significant differences between HCs and MDD patients. Meanwhile, we used the Random Forest algorithm to identify the critical discriminatory OTUs. The ROC curve analysis was used to assess the diagnostic performance of these identified differential bacterial taxa. The AUC was the evaluation index. Finally, we used the LEfSe to reveal the changes of microbial abundances at Family level and Genus level in HCs and MDD patients, respectively.
